# Differences in the muscle activities of the quadriceps femoris and hamstrings while performing various squat exercises

**DOI:** 10.1186/s13102-022-00404-6

**Published:** 2022-01-21

**Authors:** Joo-Hyun Lee, Soojin Kim, Jihye Heo, Dong-Ho Park, Eunwook Chang

**Affiliations:** 1 College of Arts and Sports, Department of Kinesiology, Inha University, 5W555B, 100 Inha-ro, Michuhol-gu, Incheon, 22212 South Korea; 2 Institute of Sports and Arts Convergence (ISAC), Inha University, W-440, 100 Inha-ro, Michuhol-gu, Incheon, 22212 South Korea

**Keywords:** Electromyography, Spanish Squat, Rectus Femoris, Vastus Lateralis

## Abstract

**Background:**

Knee injuries in the lower limbs frequently occur, and lower limb muscles need to be strengthened to reduce injuries. Activating muscles can help strengthen muscles.. This study aimed to determine the squat exercises [general squat (GS), wall squat (WS), and Spanish squat (SS)] that effectively increased muscle activity using electromyography (EMG).

**Methods:**

In this cross-sectional study, 22 participants performed three different squat exercises with EMG attached to the rectus femoris (RF), vastus lateralis (VL), vastus medialis, biceps femoris, semitendinosus, and semimembranosus. The Kruskal–Wallis H test was used to compare thigh muscle activities among the various squat exercises.

**Results:**

During SS, RF showed greater muscle activation compared to WS and GS (RF: χ2 = 21.523, *p* = 0.000, η^2^ = 0.333). VL also showed greater muscle activation during SS compared to WS (VL: χ2 = 7.101, *p* = 0.029, η^2^ = 0.109).

**Conclusions:**

The results from this study indicate that SS shows more activation in the RF and VL muscles compared to GS and WS. These findings suggest that SS can provide more muscle activation for the RF and VL muscles and will greatly help those who lack muscle activation in these muscles.

## Background

The increasing popularity of sports is also concomitant with an increase in the number of injuries. In fact, more than 55% of all sports related injuries occur in the lower limbs, and specific damage to the knee accounts for approximately 15% of all sports related injuries [1]. The knee is a biomechanical and anatomical complex joint [2] that is functionally responsible for weight loading, requires a large range of motion. In addition, the knee joint is surrounded by many ligaments and muscles that could have a potential of injury secondary to external force. The most important muscles around the knee joint are the quadriceps femoris, and exercise that increases the activation of this muscle is effective in strengthening muscles.[3]. Notably, squats are one of the most common exercises to increase lower limb muscle strength and activation [4], and muscle strength improvement is effective in preventing injuries [5]. The main knee muscles used during squat exercises are the quadriceps, hamstrings, and gastrocnemius, wherein co-contractions among these muscles improve knee stability [6] The prescription of squat exercise can improve leg, hip, and back strength [7], and is additionally a major exercise in sports performance and knee rehabilitation programs [8].

Squat exercises for improving quadriceps strength are said to be functional since they require more joint movement than other lower limb exercises, promoting functional muscle mobilization patterns [9]. Generally, three different types of squat exercise are applied in the field of rehabilitation: general squat (GS), wall squat (WS), Spanish squat (SS). During GS, the antagonistic muscles may have a significant influence on the stability of the weakened joints due to the agonist muscle co-activation [10]. However, the hamstring muscles showed weaker muscle activation during GS because these muscles are biarticular structure [11] and some researches show that weight bearing back squat training does not provide a sufficient stimulus for hamstring muscles [12, 13]. Additionally, GS can increase pressure and stress on the lumbar spine [14] and could lead to an increase in spinal injuries [15]. However, those injury risks could be reduced by performing WS with similar exercise effect of GS. Due to the benefits of WS, WS has been applied for maintaining quadriceps strength to prevention of patellofemoral pain and recovering from knee joint injuries [16]. Although WS with Swiss ball is often used to facilitate greater muscle activation [17], the location of the wall and feet altered biomechanical force on the patellofemoral joint which could potentially results in increasing patellofemoral force and reducing the effect of exercise [18]. Recently, a previous study reported that SS could be a good option to apply for managing individuals with patellofemoral pain because the exercise position provided mechanical advantages on the quadriceps muscles which could be a major role of knee joint health [19, 20]. While the hamstring also plays an important role for knee joint health, there are insufficient information regarding muscle activation in hamstring muscles during WS and SS.

Electromyography (EMG) is an instrument that can measure and collect muscle activity and widely used in sports science and sports medicine fields. EMG signals can also be used for clinical and biomedical applications [21]. Several studies have even shown EMG analyses of lower limb muscle activity during a variety of closed kinetic chain exercises such as GS [22, 23] Additionally, previous study have shown the muscle activity of quadriceps during GS and WS exercises [24]. Despite these findings, studies comparing the quadriceps and hamstring muscles during GS, WS, and SS exercises are insufficient. Therefore, the current study aimed to compare these three squat exercises to identify which exercises can effectively activate quadriceps femoris and hamstring muscles, and to provide more diverse information to rehabilitation programs or professional clinicians. Thus, we speculate that our study can be effectively performed by selecting the type of exercise and increasing muscle activation through exercises can increase muscle strength to prevent injuries. We hypothesized that SS would show the highest muscle activity when performing the various squat exercise using EMG in the quadriceps femoris, and no significant differences would be found in the hamstring muscles.

## Methods

### Study design

We used a cross-sectional design with repeated measures, wherein all participants performed three exercises in a randomized order as we recorded EMG measurements for the quadriceps and hamstring muscles activation. During the data reduction process, only 22 participants performed data analysis due to the missing data.

### Participants

A power analysis performed by G*POWER indicated 21 participants were needed with a power of 0.80, an effect size of 0.7, and an α = 0.05. A total of 25 male volunteers between the age of 20 and 30 years were recruited to participate in this study. All participants were active, which was defined as 60 min of physical activity for at least three days per week. They had no history of ACL injury, lower back pain, or lower extremity joint surgery, reported no symptoms of injury at the time of testing, and could perform the exercises without pain. However, 3 participants were excluded due to data reduction errors. The remaining 22 healthy individuals participated in the experiment until the end of the study (Table 1). The protocols for the present study were submitted to and approved by the Ethical Committee of the institution (IRB code: 213055-1A) and all participants provided their written informed consent prior to participation in this study.

**Table 1:** Participant demographics

Characteristics	Mean ± standard deviation
Age (years)	23.0 ± 2.7
Height (cm)	177.5 ± 4.7
Mass (kg)	76.1 ± 6.5
Body mass index (kg/m^2^)	24.2 ± 2.2
Godin activity questionnaire	60.5 ± 32.5

### Instrumentation

A wireless surface EMG system (Trigno Sensor System, Delsys Inc., Natick, MA, USA: interelectrode distance = 10 mm, 80 dB common mode rejection rate) was used to record lower extremity muscle activity. EMG data were sampled at 2000 Hz, and maximal voluntary isometric contractions and exercises were exported using EMG works® analysis software.

**Fig. 1:**
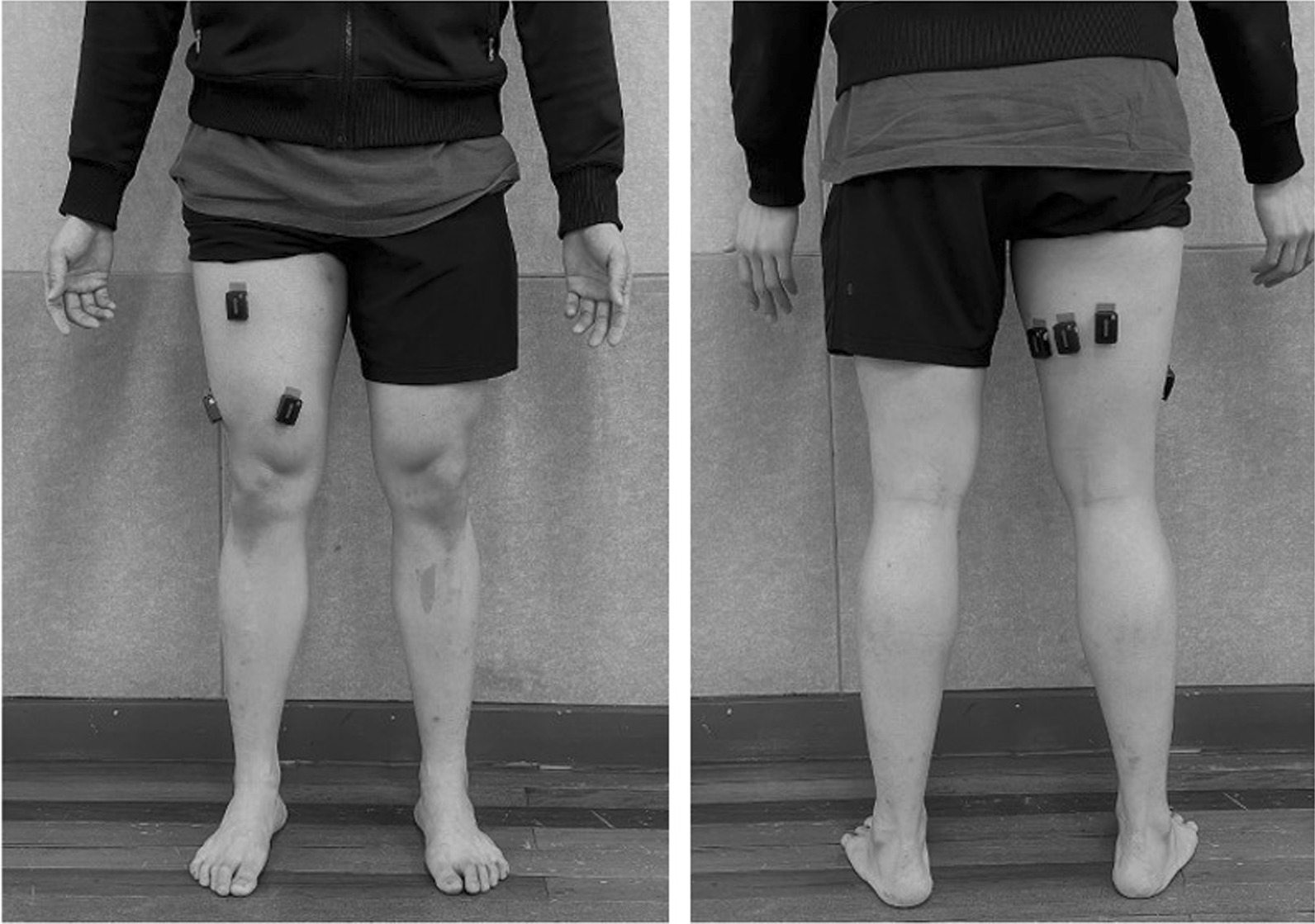
Positioning of electromyography electrode of rectus femoris (RF), vastus lateralis (VL), vastus medialis (VM), biceps femoris (BF), semitendinosus (ST), semimembranosus (SM)

Figure 1 showed the placement of the EMG electrodes on the quadriceps and hamstring. The electrodes for the rectus femoris (RF) were placed at 50% on the line from the anterior superior iliac spine (ASIS) to the superior part of the patella. For the vastus lateralis (VL), the sensor was placed 2/3 of the way on a line from the ASIS to the lateral side of the patella. While for the vastus medialis (VM), the sensor was placed at 80% on a line between the ASIS and the joint space in front of the anterior border of the medial ligament [25]. In the case of the biceps femoris (BF), the electrodes were placed halfway between the ischial tuberosity and the lateral epicondyle of the tibia [26], and the semitendinosus (ST) and semimembranosus (SM) electrodes were positioned medially, just perpendicular to the BF [27].

### Maximal voluntary isometric contractions

**Fig. 2:**
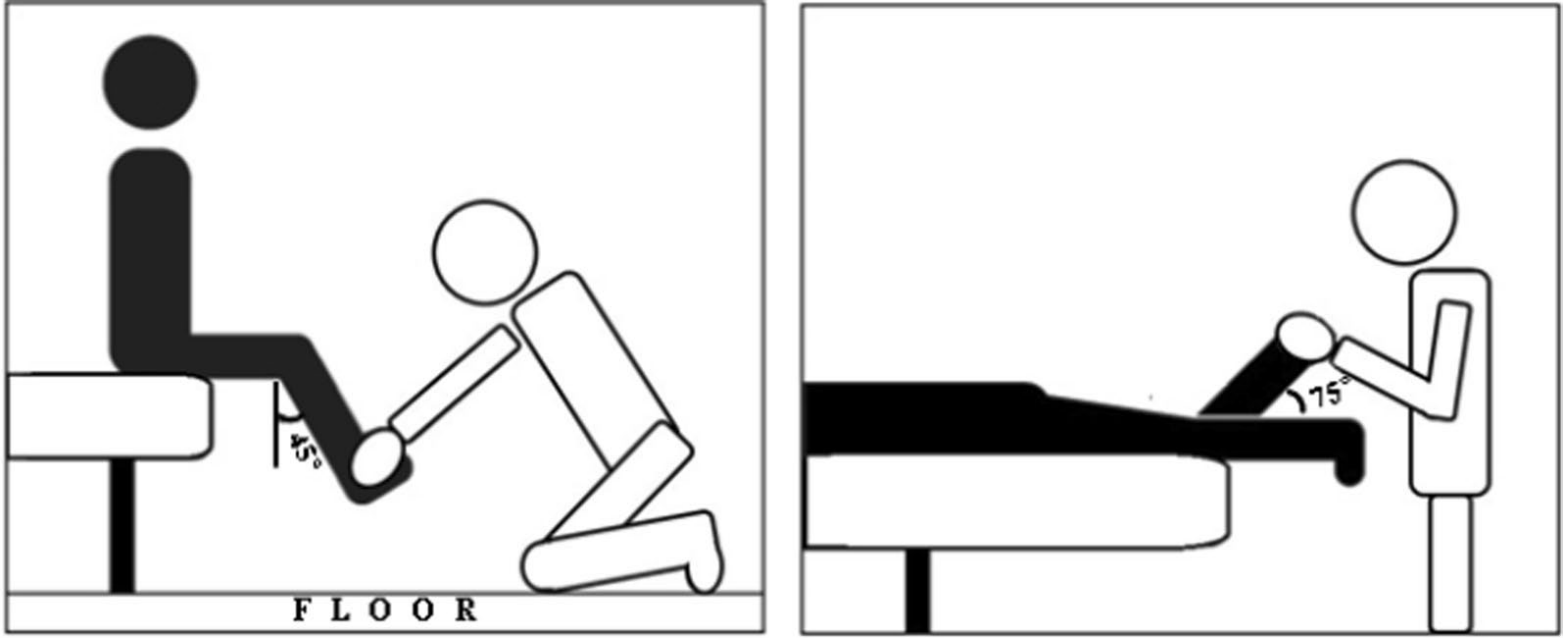
Position for performing maximal manual muscle testing (MMT) on quadriceps femoris and hamstrings

We measured the maximum voluntary isometric contractions (MVIC) using manual muscle testing (MMT). Previous study compared hand-held dynamometer and fixed dynamometer to intraclass correlation coefficient (ICC) [28] and the ICC values of knee extensor and knee flexors are 0.82 and 0.80, respectively, showing good reliability (ICC < 0.5 = poor reliability, 0.5 < ICC < 0.75 = moderate reliability, 0.75 < ICC < 0.9 = good reliability, and 0.90 < ICC = excellent reliability). So, our study proceeded MMT based on this. The testing session began with a series of two MVIC for each muscle, with 5 s maximum contractions interspersed for 60 s. The participants performed MMT of the dominant side and were measured with the knee extensors at 45° of the knee flexion [29]. The hamstring muscles were placed in a prone position [30], and the maximum resistance to the knee flexion was applied with the participant’s knee flexed to 75° (Fig. 2).

### Squat exercise

**Fig. 3:**
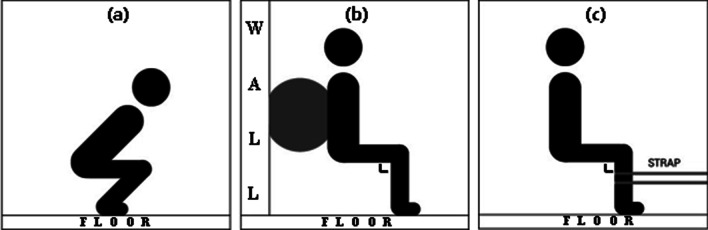
Positioning for squat exercise **a** GS, **b** WS, **c** SS

GS started without any weight while the feet were positioned shoulder-width apart and were angled approximately 15–30° laterally [31] (Fig. 3a). The depth of the GS used in this study was measured with the inguinal fold at the same level as the superior aspect of the knee [32]. WS put the Swiss ball between the wall and the lumbar spine and put both feet on the floor. The participants then descended until the torso to pelvis and knee angle were 90° [17]. Moreover, foot placement was not constrained to enable the participants to complete the exercise in a comfortable position (Fig. 3b). SS were positioned with the assistance of rigid straps placed below the knee joint, and the feet were positioned shoulder-width apart. The posture of SS is to prefer that the angle of the knee and the torso to pelvis are vertical (Fig. 3c). All these squat exercises began with the experimenter’s ‘start’ verbal sign and took a 1 s to reach the nearest position to the floor. The participants held the nearest position to floor for 3 s and returned to the original position for 1 s. This action was repeated three times and the data for each squat exercises’ isometric phase was collected and selected for analysis.

### Data reduction

Muscle activity of the quadriceps femoris and hamstrings was collected using Delsys surface electrodes for the RF, VL, VM, BF, ST, and SM on the participants’ dominant side. Data for each surface electrode were sampled at 2000 Hz and were collected using the EMG Works ® analysis software. Prior to each test session, the participants’ MVIC for each muscle was obtained using resistance isometric contractions to ensure that the data could be normalized. The MVIC data and raw data were then transferred to EMG Works ® analysis software, replaced with Excel, and were sent to MATLAB (MathWorks R2020b). Then, the EMG signals were band-pass filtered (10–500 Hz, 4th order Butterworth) and smoothed using the RMS values and a 1 s moving-window function. All data were filtered and smoothed, and peak data were selected and normalized based on the maximum EMG signals recorded during maximal voluntary contractions, presenting these values as %MVIC.

### Statistical analysis

The data were analyzed using the sub-problems of the study. To determine the relationship between various squats and quadriceps muscle activities, correlation analysis was used to reveal the level and direction of the relationship between each thigh muscle (RF, VL, VM, BF, ST, and SM). Based on the Shapiro–Wilk test, the research data were not normally distributed (*p* < 0.05), and the variances were not homogeneous. Therefore, the Kruskal–Wallis H test was used to analyze the data, and the Mann–Whitney U test was used to determine which groups had significant differences in the Kruskal–Wallis H test results. The effect size was calculated and expressed by eta-square based on H-statistics. During the statistical analysis, the outliers in BF and SM were excluded.

## Results

**Fig. 4:**
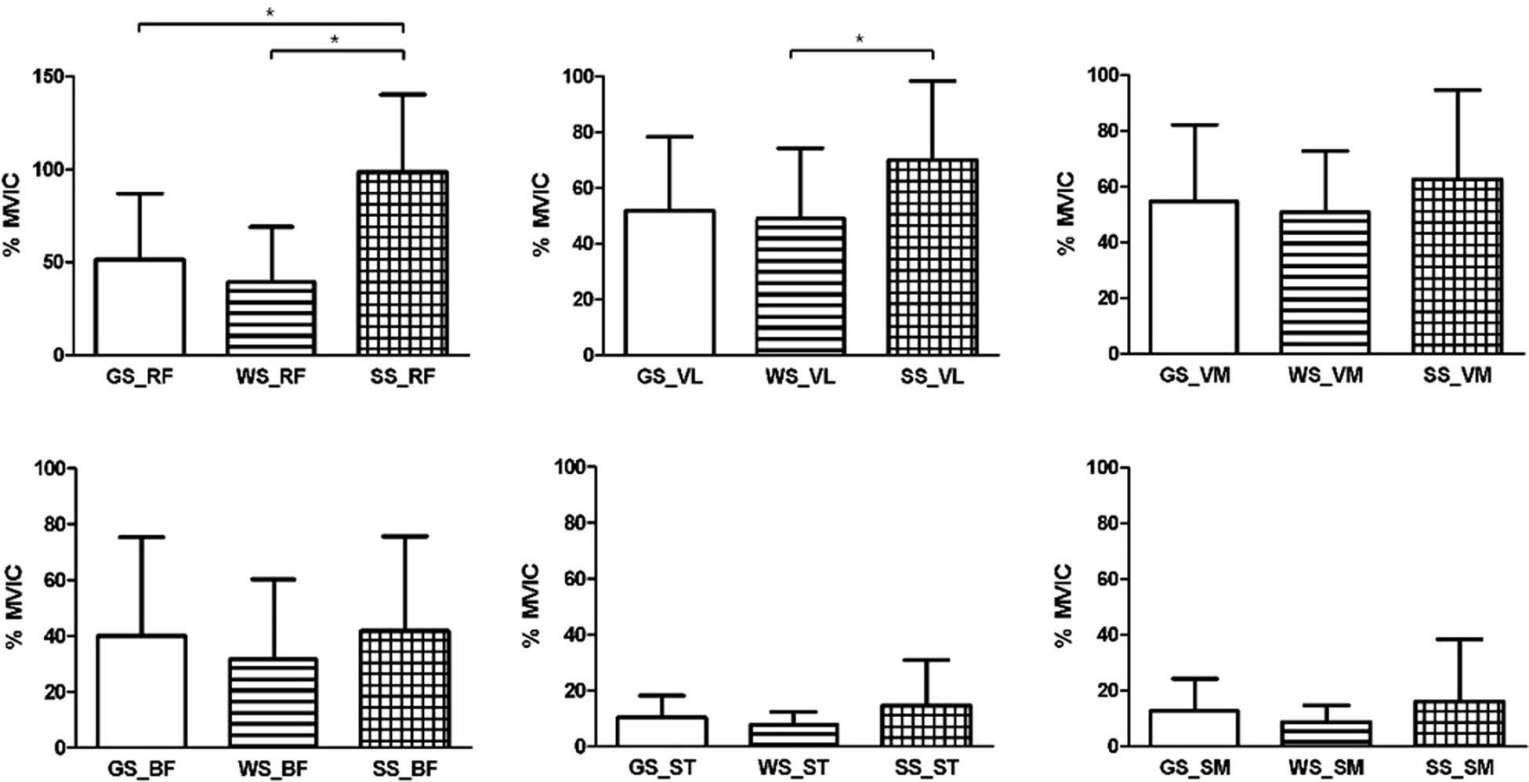
Mean (± SD) normalized rectus femoris (RF), vastus lateralis (VL), vastus medialis (VM), biceps femoris (BF), semitendinosus (ST) and semimembranosus (SM) EMG for general squat (GS), wall squat (WS) and Spanish squat (SS)

The Kruskal–Wallis H test showed the mean accuracy values in terms of different squat exercises (GS, WS, SS), with RF (H (2) = 21.523, *p* = 0.000 and η^2^ = 0.333). In this regard, the SS value (Median = 91.5) was higher compared to the WS (Median = 29.5) and GS (Median = 36.5) values. In terms of VL (H (2) = 7.101, *p* = 0.029 and η^2^ = 0.109), the SS (Median = 63) was also higher compared to the WS (Median = 47.5) and GS (Median = 48). Post-hoc Mann–Whitney tests using a Bonferroni-adjusted alpha level of 0.017 (0.05/3) were used to compare each group, showing a significant difference between the RF (between GS and SS, and WS and SS) and VL (between WS and SS) values. In addition, RF is a large effect size, and VL shows a medium effect size (Fig. 4, Table 2).

**Table 2:** Results of the Kruskal–Wallis H test on the mean accuracy values according to the different squat exercises (general squat (GS), wall-squat (WS), Spanish squat (SS))

Muscles	Group	n	Mean rank	χ2	*p*	Difference	η^2^
Rectus femoris	GS	22	29.20	21.523	.000*	GS – SS WS – SS	.333
	WS	22	22.75				
	SS	22	48.55				
Vastus lateralis	GS	22	29.98	7.101	.029*	WS – SS	.109
	WS	22	28.18				
	SS	22	42.34				
Vastus medialis	GS	22	32.05	2.347	.309		.037
	WS	22	29.98				
	SS	22	38.48				
Biceps femoris	GS	21	33.29	2.060	.357		.057
	WS	22	27.64				
	SS	20	35.45				
Semitendinosus	GS	22	34.23	3.392	.183		.026
	WS	22	27.86				
	SS	22	38.41				
Semimembranosus	GS	22	35.00	1.656	.437		.021
	WS	22	28.80				
	SS	21	35.31				

* P < 0.05 significant differences exist between at least one of the three groups. η^2^ ≥ 0.01 small, η^2^ ≥ 0.06 medium, η^2^ ≥ 0.14 large.

## Discussion

This current study sought to compare all the three squat exercises (GS, WS, and SS) and their respective neuromuscular activity in the quadriceps and hamstring muscles. The significant findings of this study showed that there was greater muscle activity in the RF when performing SS compared to GS and WS. The VL muscle also showed a significant difference between SS and WS. RF muscle show a large effect size of 0.333, and VL muscle show a medium size effect of 0.109. In contrast, the VM, BF, ST, and SM muscles did not show significant differences among the three exercises.

Muscle activation of RF muscle showed higher activity SS than GS. Other study, increasing hip flexion torque contributes hamstring muscles, gluteus maximus, and adductor magnus necessary for hip extensor, thus the RF muscle activation is increasing. [6]. During squat exercises with the torso more vertical to the pelvis probably more effective as a knee extensor for RF muscle because RF muscle is longer when the torso is raised vertically than when the torso is tilted [6]. Our study, we can see that the angle of the torso to the pelvis was more upright in SS than in GS. Therefore, the muscle activation of the RF muscle can be seen as higher during SS.

Additionally, the RF and VL muscles showed different muscle activation between the SS and WS. In order to successfully perform the SS, it is essential to have proper torso to pelvis position and knee joint angle. A previous investigation showed that upright torso position and 90 degrees of knee joint angle during SS resulted in increasing lever arm distance between center of mass of the body and the point of ground reaction force applied [20].As the angle of the upper body gradually approached 90 degrees, the distance between the lever arm increased thus, the external knee extensor moment also increased [20]. These biomechanical characteristics of SS could influence quadriceps muscle activation. The WS and SS showed relatively similar knee and torso to pelvis angles, but the WS showed lower muscle activation than SS. Recent study showed that during WS the torque of knee is the distance between knee and the center of body mass multiplied by the body mass, eliminating the force pushing the wall [33]. However, unlike Biscarini’s research [33], SS did not eliminate the force of pushing the wall, therefore the uneliminated force is placed on the thighs with a higher load than WS. For this reason, higher torque will be shown in the knee joint during SS. This could have the RF and VL muscles are larger muscle activation during SS than WS.

VM has been considered to have an important role for patella stabilization. A previous study reported that VM assists the RF function during knee extension and helps the patella to maintain the center [34]. Clinically, the VM was associated with reduced knee pain patients with osteoarthritis of the knee joint [35]. Thus, recent studies are focused primarily on the VM muscle. Although VM provides essential functions on the knee joint, the RF muscle is also in charge of gait control, reducing its contribution to produce increased knee flexion and inappropriate foot position at ground contact [36]. Moreover, VL muscle is a synergistic muscle to stabilize the patella during knee extension [37]. Thus, the weakening of the RF and VL muscles may weaken the knee joint and trigger pain around patellar. The current study showed that the RF and VL muscles were largely activated during 90 degrees isometric SS. These findings could suggest that SS can be used for rehabilitation purposes in injured patients, preventing knee joint problems and patellar diseases by strengthening the aforementioned muscles. Accordingly, clinical specialists will need to develop a variety of exercise methods and treatments using SS.

On the other hand, the hamstring muscles did not show significant changes during the squat exercises. A previous study indicated weaker muscle activation of the hamstring muscles during the back squat exercise since there was little change in the muscle length [38]. Thus, the various squat exercises we performed in our study showed low muscle activity as the length of the hamstring did not change because the exercise eventually measured by isometric contraction and modified the squat motion. Furthermore, in another study, squat was not sufficient to stimulate the hamstring muscles [12]. Since our study utilized non-weight bearing, it is speculated that the hamstring muscles were not sufficient to stimulate muscle activation.

Despite these findings, this study had several limitations. First, the low participant number in this study (n = 22) may have been underpowered because some participants failed during exercise. Second, standard squat positions were recommended for all the conditions during this study; however, the pelvic and lumbar angles during squats were often inaccurately reflected owing to the physical differences in the participants which might have affected muscle activation. Third, our study measured the experiment without motion capture equipment, there may be errors in the accuracy of the squat exercises motion. Fourth,EMG analysis is a valuable instrument for evaluating muscle activity, but it can also affect the results of the user’s muscle anatomy understanding, EMG attachment proficiency, and differences in the shape of each participant’s muscles. Finally, this study evaluated EMG activity during various squat exercises of the quadriceps and hamstrings, which are important for knee injury rehabilitation and prevention. However, many other lower limb muscles are also important for maintaining lower limb stability.

## Conclusions

The current study compared the muscle activation of the quadriceps and hamstring muscles during squat exercises. The RF muscle showed activation differences in the GS-SS and WS-SS comparisons, and VL muscles showed activation differences in the WS-SS comparison. According to the results from the current study, SS exhibited greater muscle activation on RF and VL compared to GS and WS, it could be a potential option to include SS for prescribing exercise or designing rehabilitation programs. Further studies are warranted to investigate the muscle activity of the lower limbs and potential rehabilitation approaches for injured patients.

## Data Availability

The datasets used and/or analyzed during the current study are available from the corresponding author (EC) on reasonable request. The data sets are not publicly available due to restrictions imposed by Inha University Institutional Review Board (IRB NUMBER: 213055-1A).

## Abbreviations

GS: General squat
WS: Wall squat
SS: Spanish squat
RF: Rectus femoris
VL: Vastus lateralis
VM: Vastus medialis
BF: Biceps femoris
ST: Semitendinosus
SM: Semimembranosus
EMG: Electromyography
